# Antenatal HIV screening: results from the National Perinatal Survey, France, 2016

**DOI:** 10.2807/1560-7917.ES.2019.24.40.1800573

**Published:** 2019-10-03

**Authors:** Thi-Chiên Tran, Josiane Pillonel, Françoise Cazein, Cécile Sommen, Camille Bonnet, Béatrice Blondel, Florence Lot

**Affiliations:** 1 Santé publique France, French national public health agency, Saint-Maurice, France; 2 Université de Paris, CRESS, INSERM, INRA, Paris, France

**Keywords:** antenatal HIV screening, antenatal care, pregnant women, national perinatal survey, maternal characteristics

## Abstract

**Background:**

Universal antenatal HIV screening programmes are an effective method of preventing mother-to-child transmission.

**Aims:**

To assess the coverage and yield of the French programme on a nationally representative sample of pregnant women, and predictive factors for being unscreened or missing information on the performance/ result of a HIV test.

**Methods:**

Data came from the medical records of women included in the cross-sectional 2016 French National Perinatal Survey. We calculated odds ratios (OR) to identify factors for being unscreened for HIV and for missing information by multivariable analyses.

**Results:**

Of 13,210 women, 12,782 (96.8%) were screened for HIV and 134 (1.0%) were not; information was missing for 294 (2.2%). HIV infection was newly diagnosed in 19/12,769 (0.15%) women screened. The OR for being unscreened was significantly higher in women in legally registered partnerships (OR: 1.3; 95% CI: 1.1–1.6), with 1–2 years of post-secondary schooling (OR: 1.6; 95% CI: 1.2–2.1), part-time employment (OR: 1.4; 95% CI: 1.1–1.8), inadequate antenatal care (OR: 1.9; 95% CI: 1.5–2.4) and receiving care from > 1 provider (OR: 1.8; 95% CI: 1.1–2.8). The OR of missing information was higher in multiparous women (OR: 1.4; 95% CI: 1.2–1.5) and women cared for by general practitioners (OR: 1.4; 95% CI: 1.1–1.9).

**Conclusions:**

The French antenatal HIV screening programme is effective in detecting HIV among pregnant women. However, a few women are still not screened and awareness of the factors that predict this could contribute to improved screening levels.

## Introduction

An important route for human immunodeficiency virus (HIV) infection is via mother-to-child transmission (MTCT), which can occur during pregnancy, delivery or through breastfeeding. MTCT is preventable through effective public health measures, including antenatal HIV screening and combined antiretroviral therapy (cART). Since 1993, France has included universal HIV testing in its nationwide antenatal programme [1]. Healthcare professionals are supposed to systematically propose an HIV test to all pregnant women during the first trimester of pregnancy and at least one additional test at the beginning of the third trimester for women at high risk, i.e. injecting drug users, sex workers, as well as those with HIV-infected sex partners, new or multiple sex partners during pregnancy [1-3]. The goal is to test 100% of women unless they have been tested recently. HIV testing (fourth-generation ELISA) is free in all public or private laboratories if women have a prescription from healthcare professionals [2]. The antenatal programme also recommends eight medical visits and three ultrasound examinations for low-risk, full-term pregnancies [1], which are mostly covered by National Health Insurance Fund in the first months of pregnancy and completely covered from the sixth month of pregnancy, except in the private sector where women have to pay extra costs.

A universal antenatal HIV screening programme enables the best possible perinatal antiretroviral therapy (ART) management for HIV-infected mothers and children and is the most effective method of preventing MTCT [4-8]. Such programmes and subsequent perinatal cART have reduced MTCT rates markedly in Europe: in Sweden, from 24.7% in the period 1985–1993 to 0.6% in the period 1999–2003 [5]; in France, from 20% before 1994 to 1.5% in the period 1994–2004 [4]; and in the United Kingdom (UK), from 2.1% in the period 2000–2001 to 0.5% in the period 2010–2011 [8].

Nonetheless, MTCT of HIV infection continues to occur in high-income countries because some women still do not receive or refuse the opportunity for HIV testing, or are diagnosed too late to be able to benefit from cART and can, therefore, transmit HIV [7-12]. A study of children born with perinatal HIV infection from 2006 to 2013 in the UK showed that of the 108 mothers of these children, 67 (60%) were undiagnosed at delivery [10]. Among them, 28 (41.8%) had not had antenatal HIV testing, 23 (34.3%) seroconverted after an earlier HIV-seronegative test result during pregnancy, 11 (16.4%) had a problem with the test because of processing/reporting errors or late antenatal booking, and information was missing for five women. Similar factors have also been described for perinatally HIV-infected children born in a Parisian hospital from 2006 to 2012 [9].

Mandatory HIV reporting began in France in 2003, with MTCT accounting for 1.0% of the 73,481 new HIV diagnoses recorded up to 2017. Among 8,463 new diagnoses in women between 2008 and 2017 with a known CD4^+^ T-cell count, 5,311 (62.8%) were in women aged 15–39 years. Of these women of childbearing age, 2,731 (51.4%) were diagnosed late (CD4^+^ < 350 cells/mm^3^ at diagnosis).

Interviews after childbirth during the 2010 French National Perinatal Survey (2010 NPS) showed that 68.9% of 13,891 participating women said that they were tested for HIV during pregnancy; 7.5% did not know if they had been tested or not; 8.0% said that they had not been tested because no healthcare provider had proposed it; 4.5% were not tested because they had had an HIV-negative test result shortly before the pregnancy; 4.6% were not tested for another reason; 1.0% refused testing, and; 5.4% had missing data about antenatal HIV screening [13].

Using data from the 2016 French National Perinatal Survey (2016 NPS), which collected information on HIV screening from medical records, we aimed to assess the present performance of the antenatal HIV screening programme and identify predictive factors associated with being unscreened and missing information about the mother’s testing.

## Methods

### Study design

The French National Perinatal surveys are cross-sectional studies designed to monitor perinatal health indicators and to guide health policies [13,14]. They include all live births and stillbirths with a gestational age ≥ 22 weeks or a birth weight ≥ 500 g occurring in all public and private maternity units over a 1-week period. The 2016 NPS [14] was performed in March 2016.

Three questionnaires were used to collect patient and institutional characteristics: (i) a form completed by investigators during interviews with women in post-partum wards to obtain maternal socio-demographic characteristics and information about antenatal care and delivery (74 questions); (ii) a medical questionnaire completed by investigators using information about antenatal HIV testing, medical, obstetric and perinatal care characteristics extracted from the mothers’ medical records (72 questions); (iii) a form where heads of maternity units described the principal institutional characteristics, e.g. the staff and the organisation of antenatal visits, birth rooms and postnatal care (59 questions). No information was collected on sexual behaviour or intravenous drug use.

All investigators, most of them midwives, were trained how to conduct the survey.

### Categories of maternal characteristics and type of antenatal care

Legally registered partnerships include those in legal marriages or with civil unions, which are known in France as civil solidarity pacts. Single mothers were considered women who did not live with their partner during pregnancy. Women whose monthly household income was less than EUR 1,500 were defined as low-income, EUR 1,500–4,000 as intermediate-income, and more than EUR 4,000 as high-income.

In France, women can choose the health professional who will monitor their pregnancy. The main healthcare provider in the first 6 months of pregnancy was classified into six categories: (i) obstetrician and/or gynaecologist in private practice; (ii) obstetrician/gynaecologist in public practice; (iii) general practitioner (GP) in the private sector; (iv) midwife in the public sector; (v) midwife in the private sector; and (iv) more than one healthcare provider if women were cared for by different doctors or midwives in Maternal and Child Health centres or by more than one medical professional in different practices.

Women with inadequate antenatal care were defined as those who had fewer than the minimum number of antenatal visits or ultrasound examinations recommended in France for gestational age at delivery.

For the analysis, France was divided into 13 administrative regions: 12 regions of mainland France (Corsica was included in the Provence-Alpes-Côte d’Azur region) and the overseas region (including Guadeloupe, Guyana, Martinique, Mayotte and La Réunion).

### Outcomes measured

The medical questionnaire completed by investigators allowed for six different responses about HIV testing during pregnancy: (i) Yes, HIV-seronegative; (ii) Yes, HIV-seropositive; (iii) No, because HIV-positive status was known before pregnancy; (iv) No, because of a recent negative test result before pregnancy; (v) No, for other reasons, e.g. late antenatal care or test refusal, and; (vi) No information regarding the performance or the result of a test.

The three possible antenatal HIV screening outcomes were: (i) screened if women were tested for HIV during pregnancy or knew their HIV-infection before pregnancy (response i to iii above); (ii) unscreened if women were not tested during pregnancy (response iv or v above); and (iii) missing information (response vi above).

In our analysis, we excluded women for whom investigators did not respond to the question about HIV testing in in the medical questionnaire.

### Statistical analysis

We selected 17 covariables considered to be possible confounders of the outcome from the literature [7,8,10,11,14-16].

In the univariate analyses, the differences between HIV screening groups were assessed by Kendall tests for continuous variables and Pearson’s chi-squared tests for qualitative variables. Maternal age was tested both as a continuous variable and a categorical variable according to the following age groups: < 20 years, 20–24 years, 25–29 years, 30–34 years, 35–39 years, 40–44 years and ≥ 45 years.

In the multivariable analyses, we started by verifying the missing-data assumptions for the covariables. Then, we used a multiple imputation (MI) procedure by fully conditional specification methods [17] to handle missing covariable values and minimise the bias they could cause. Overall, 116 variables with missing data < 20% were selected in the MI model to generate seven imputed datasets [17].

Covariables with a p value < 0.2 in the univariate analyses were adjusted in stepwise multinomial logit models [18] that were applied to the imputed datasets. Only variables with a p value < 0.05 were retained in the final model to determine predictive factors for the odds of unscreened or missing information outcomes.

Sensitivity analyses compared the results obtained from the same final model across the imputed datasets and the complete database before imputation (raw database).

Based on the plot of the standardised residuals and robust regressions, diagnostics to check the multiple analyses were performed to ensure the quality and the reliability of the results of each multivariate model.

The statistical associations of factors associated with HIV screening are expressed as odds ratios (OR) with their corresponding 95% confidence intervals (CI).

The statistical analyses were performed with SAS software version 9.4 (SAS Inc., Cary, North Carolina, United States (US)).

### Ethical statement

The survey was approved by the National Council on Statistical Information, the French Data Protection Authority and the ethics committee of the National Institute for Health and Medical Research. The approval numbers respectively were 2016X703SA, 915197 and IRB00003888 no. 14–191.

## Results

### Study participation

Overall, 4 of 517 (0.8%) maternity units in France refused participation in the 2016 NPS, corresponding to about 120 missing births. In the 513 participating units, 14,142 infants were born to 13,894 women during the survey period. The medical questionnaire was available for 13,241 women (95.3%) aged 17 to 49 years (Figure).

**Figure:**
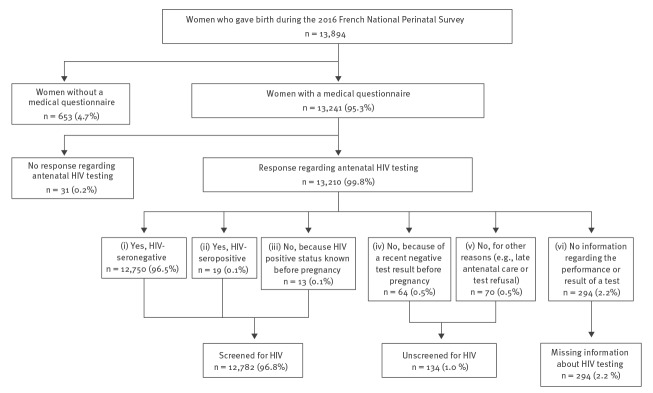
Flowchart of antenatal HIV screening study population, National Perinatal Survey, France, 2016

Among the 653 women without a medical questionnaire, 206 (31.5%) refused to participate in the survey, 227 (34.8%) were not interviewed because of concerns raised by the data protection committee (minors, women with stillbirths, or planned surrenders for adoption), and 220 (33.7%) could not participate because of language barriers, health problems or early discharge from the maternity unit.

Among the 13,241 women with a medical questionnaire, 31 (0.2%) were excluded because no response was given to the question regarding HIV testing. Maternal socio-demographic characteristics and maternity unit characteristics did not differ between the women excluded and those included, overall or by screening outcome category, i.e. screened, unscreened and missing information.

Missing data accounted for 0%–8% of the values for 16/17 covariables, reaching 12% in the remaining covariate (partner’s occupational status).

### HIV screening

Among the 13,210 women included in our analysis, antenatal HIV screening was performed for 12,782 (96.8%), not performed for 134 (1.0%) and information was missing for 294 (2.2%) (Figure).

Among the 134 unscreened women, 64 (47.8%) were not tested because of a recent negative HIV test result before pregnancy and 70 (52.2%) for other reasons (e.g. late antenatal care, test refusal).

Of the 12,782 screened, 13 were not tested because they were already known to be HIV-positive before pregnancy. HIV infection was newly diagnosed in 19 (0.15%) of 12,769 women tested during pregnancy.

### Maternal characteristics according to HIV screening

In univariate analyses, among the demographic and social characteristics associated with HIV screening with a p value < 0.05, the proportion of women screened was higher among those not in a legally registered partnership (97.2%), born outside France (≥ 97.6%), unemployed (97.2%) or employed full-time (97.0%), and those who had a low-income (97.6%) or a high-income (97.2%) (Table 1). The percentage of unscreened women was higher among those in a legally registered partnership (1.2%) or with part-time employment (1.7%) or with an intermediate-income (1.1%). We also found higher missing information outcomes for women in a legally registered partnership, born in France, with part-time employment or with an intermediate-income.

**Table 1:** Univariate analysis of antenatal HIV screening according to maternal characteristics, National Perinatal Survey, France, 2016 (n = 13,210)

Variable		Overall participants (n = 13,210)	HIV screening outcome						p value^a^
			Unscreened (n = 134)		Missing information (n = 294)		Screened (n = 12,782)		
		n	n	% or Mean ± SD	n	% or Mean ± SD	n	% or Mean ± SD	
Age at delivery	Years	13,200	133	30.0 ± 5.1	293	30.7 ± 4.9	12,774	30.2 ± 5.2	0.18
Single mother	No	11,140	110	1.0	254	2.3	10,776	96.7	0.13
	Yes	1,218	9	0.7	18	1.5	1,191	97.8	
Legally registered partnership	No	5,315	38	0.7	112	2.1	5,165	97.2	0.04
	Yes	7,015	81	1.2	159	2.3	6,775	96.6	
Country of birth	France	9,981	100	1.0	238	2.4	9,643	96.6	0.03
	Sub-Saharan Africa	683	5	0.7	6	0.9	672	98.4	
	Other countries	1,717	14	0.8	28	1.6	1,675	97.6	
Education level	Did not complete high school	2,815	28	1.0	52	1.8	2,735	97.2	0.16
	Completed high school	2,691	23	0.9	58	2.2	2,610	97.0	
	1–2 years post-secondary school	2,346	34	1.4	55	2.3	2,257	96.2	
	3–4 years post-secondary school	2,197	15	0.7	49	2.2	2,133	97.1	
	≥ 5 years post-secondary school	2,115	16	0.8	53	2.5	2,046	96.7	
Maternal occupational status	Unemployed	3,741	33	0.9	71	1.9	3,637	97.2	0.007
	Employed part-time	1,795	30	1.7	46	2.6	1,719	95.8	
	Employed full-time	6,538	53	0.8	144	2.2	6,341	97.0	
Partner’s occupational status	Unemployed	1,617	12	0.7	24	1.5	1,581	97.8	0.06
	Employed	9,956	99	1.0	234	2.4	9,623	96.7	
Household income	Low	2,459	20	0.8	38	1.5	2,401	97.6	0.04
	Intermediate	7,528	82	1.1	184	2.4	7,262	96.5	
	High	2,145	16	0.7	45	2.1	2,084	97.2	
Health insurance plan at the beginning of pregnancy	None	300	5	1.7	3	1.0	292	97.3	0.24
	UMC or SMA	1,721	13	0.8	32	1.9	1,676	97.4	
	General health insurance	10,343	101	1.0	235	2.3	10,007	96.8	
Parity	Primiparous	5,514	44	0.8	85	1.5	5,385	97.7	< 0.0001
	Multiparous	7,696	90	1.2	209	2.7	7,397	96.1	
Preconceptional medical visit	No	8,040	76	0.9	169	2.1	7,795	97.0	0.50
	Yes	4,262	43	1.0	103	2.4	4,116	96.6	
Psychological status during pregnancy	Good or fairly good	11,055	108	1.0	250	2.3	10,697	96.8	0.38
	Fairly bad or bad	1,271	10	0.8	22	1.7	1,239	97.5	
Gestational diabetes	No	11,729	112	1.0	273	2.3	11,344	96.7	0.10
	Yes	1,433	16	1.1	21	1.5	1,396	97.4	
Inadequate antenatal care	No	12,662	121	1.0	283	2.2	12,258	96.8	0.005
	Yes	548	13	2.4	11	2.0	524	95.6	
Main healthcare provider	Obstetrician/gynaecologist (private)	6,124	43	0.7	154	2.5	5,927	96.8	< 0.0001
	Obstetrician/gynaecologist (public)	2,029	24	1.2	34	1.7	1,971	97.1	
	General practitioner	826	16	1.9	35	4.2	775	93.8	
	Midwife (public)	1,784	13	0.7	18	1.0	1,753	98.3	
	Midwife (private)	1,081	13	1.2	26	2.4	1,042	96.4	
	More than one healthcare provider	494	9	1.8	4	0.8	481	97.4	

SD: standard deviations; SMA: State Medical Aid; UMC: Universal Medical Coverage.

^a^ p values are referring to differences between the three screening outcomes.

Among the obstetric characteristics, parity affected outcome most strongly. Unscreened and missing information outcomes were more frequent among multiparous than primiparous women (1.2% vs 0.8% and 2.7% vs 1.5%, respectively, p value < 0.0001). In all, 58.3% of the participants were multiparous and accounted for 67.2% (n = 90) of the 134 unscreened women and 13 of 19 new HIV diagnoses during pregnancy.

The proportion of women with unscreened and missing information outcomes also differed significantly according to antenatal care (p value ≤ 0.005). Women with inadequate antenatal care had a higher rate of unscreened outcomes (2.4% vs 1.0%) and a lower rate of missing information (2.0% vs 2.2%) than those without inadequate antenatal care. Both outcomes were also highest among women cared for by a GP compared with those cared for by another healthcare provider in the first 6 months of pregnancy (1.9% vs 0.7–1.8% for unscreened and 4.2% vs 0.8–2.5% for missing information). The second-highest rate of unscreened outcomes and the lowest rate of missing information outcomes were found in women receiving care from more than one healthcare provider (1.8% and 0.8%, respectively). The lowest rate of unscreened outcomes was found in women receiving care from a public midwife (0.7%) or a private obstetrician/gynaecologist (0.7%).

We found no association (p value 0.05–0.2) between the outcome and maternal age as a continuous variable, single mothers, education level, partner’s occupation, or gestational diabetes.

The outcome did not differ (p > 0.2) according to maternal age as a categorical variable (data not shown), type of health insurance, preconceptional medical visit, or any of psychological status during pregnancy.

### Maternity unit characteristics according to HIV screening

The proportions of unscreened and missing information outcomes differed substantially according to the region where the maternity unit was located but not according to its size. Specifically, unscreened and missing information outcomes were lowest, respectively, among women who gave birth in maternity units of the Paris (0.3% and 0.4%), Normandie (0.2% and 1.2%) and Overseas (0.4% and 0.1%) regions and highest in the Pays de la Loire (3.4% and 9.1%) and Grand-Est (1.9% and 3.4%) regions (Table 2).

**Table 2:** Univariate analysis of antenatal HIV screening according to maternity unit characteristics, National Perinatal Survey, France, 2016 (n = 13,210)

Variable		Overall participants (n = 13,210)	HIV screening outcome						p value^a^
			Unscreened (n = 134)		Missing information (n = 294)		Screened (n = 12,782)		
		n	n	%	n	%	n	%	
Location of maternity unit, region	Paris region^b^	3,013	8	0.3	13	0.4	2,992	99.3	< 0.0001
	Grand-Est	1,040	20	1.9	35	3.4	985	94.7	
	Nouvelle-Aquitaine	924	8	0.9	32	3.5	884	95.7	
	Bourgogne-Franche-Comté	458	3	0.7	14	3.1	441	96.3	
	Bretagne	586	15	2.6	12	2.0	559	95.4	
	Centre-Val-de-Loire	465	12	2.6	8	1.7	445	95.7	
	Occitanie	956	6	0.6	36	3.8	914	95.6	
	Hauts-de-France	1,209	8	0.7	21	1.7	1,180	97.6	
	Normandie	649	1	0.2	8	1.2	640	98.6	
	Provence-Alpes-Côte d’Azur^c^	841	12	1.4	13	1.5	816	97.0	
	Pays de la Loire	770	26	3.4	70	9.1	674	87.5	
	Auvergne-Rhône-Alpes	1,574	12	0.8	31	2.0	1,531	97.3	
	Overseas^d^	696	3	0.4	1	0.1	692	99.4	
Size of maternity unit, annual deliveries	< 500	345	4	1.2	10	2.9	331	95.9	0.76
	500–999	1,879	21	1.1	42	2.2	1,816	96.6	
	1,000­1,499	2,076	16	0.8	47	2.3	2,013	97.0	
	1,500–1,999	1,954	19	1.0	51	2.6	1,884	96.4	
	2,000–3,499	4,658	45	1.0	102	2.2	4,511	96.8	
	≥ 3,500	2,247	29	1.3	42	1.9	2,176	96.8	

^a^ p values are referring to differences between the three screening outcomes.

^b^ The Île-de-France region is known as the Paris region.

^c^ The region of Corsica is included in the Provence-Alpes-Côte d’Azur region.

^d^ Guadeloupe, Guyana, Martinique, Mayotte and La Réunion were grouped together as the Overseas region.

### Predictive factors for odds of unscreened or missing information outcomes

In the multivariate analysis, several factors were each independently associated with the odds of being unscreened including; legally registered partnership, education level, occupational status, inadequate antenatal care, healthcare provider and maternity region (Table 3). Parity, healthcare provider and maternity region were each independently associated with the odds that information about HIV screening was missing (Table 3).

**Table 3:** Adjusted odds ratios of being unscreened for HIV and missing information about an HIV test, National Perinatal Survey, France, 2016 (n  = 13,210)

Variable		HIV screening outcome^a^	
		Unscreened vs screened	Missing information vs screened
		aOR (95% CI)	aOR (95% CI)
Legally registered partnership	No	Reference	Reference
	Yes	1.29 (1.08–1.55)	0.95 (0.84–1.07)
Education level	Did not complete high school	0.99 (0.71–1.38)	0.79 (0.62–1.00)
	Completed high school	0.95 (0.67–1.35)	0.97 (0.79–1.19)
	1–2-years post-secondary school	1.56 (1.19–2.06)	1.04 (0.84–1.29)
	3–4-years post-secondary school	0.74 (0.50–1.09)	1.04 (0.83–1.29)
	≥ 5-years post-secondary school	Reference	Reference
Maternal occupational status	Unemployed	Reference	Reference
	Employed part-time	1.44 (1.13–1.84)	1.06 (0.87–1.30)
	Employed full-time	0.82 (0.64–1.04)	0.96 (0.82–1.13)
Parity	Primiparous	Reference	Reference
	Multiparous	1.14 (0.97–1.34)	1.37 (1.23–1.54)
Main healthcare provider	Obstetrician/gynaecologist (private)	0.65 (0.49–0.87)	1.28 (1.06–1.53)
	Obstetrician/gynaecologist (public)	1.09 (0.78–1.53)	0.81 (0.61–1.07)
	General practitioner	1.21 (0.80–1.82)	1.43 (1.06–1.92)
	Midwife (public)	Reference	Reference
	Midwife (private)	0.98 (0.64–1.51)	1.05 (0.77–1.43)
	More than one healthcare provider	1.75 (1.10–2.78)	1.13 (0.75–1.69)
Inadequate antenatal care	No	Reference	Reference
	Yes	1.89 (1.47–2.44)	1.20 (0.91–1.57)
Location of maternity units	Paris region^b^	Reference	Reference
	Grand-Est	2.43 (1.60–3.69)	2.00 (1.45–2.76)
	Nouvelle-Aquitaine	1.11 (0.61–2.02)	2.05 (1.47–2.85)
	Bourgogne-Franche-Comté	0.73 (0.29–1.86)	1.88 (1.19–2.98)
	Bretagne	3.22 (2.02–5.13)	1.14 (0.70–1.86)
	Centre-Val-de-Loire	3.13 (1.88–5.19)	0.98 (0.55–1.76)
	Occitanie	0.78 (0.40–1.53)	2.12 (1.54–2.92)
	Hauts-de-France	0.75 (0.41–1.36)	1.02 (0.69–1.50)
	Normandie	0.17 (0.03–0.80)	0.72 (0.40–1.28)
	Provence-Alpes-Côte d’Azur ^c^	1.63 (0.98–2.70)	0.84 (0.53–1.35)
	Pays de la Loire	4.06 (2.73–6.04)	5.24 (3.98–6.88)
	Auvergne-Rhône-Alpes	0.89 (0.54–1.46)	1.11 (0.80–1.55)
	Overseas^d^	0.30 (0.11–0.80)	0.07 (0.01–0.32)

CI: confidence interval; aOR: adjusted odds ratio.

^a^ Screened (n = 12,782); unscreened (n = 134); missing information (n = 294).

^b^ The Île-de-France region is known as the Paris region.

^c^ The region of Corsica is included in the Provence-Alpes-Côte d’Azur region.

^d^ Guadeloupe, Guyana, Martinique, Mayotte and La Réunion were grouped together as the Overseas region.

The association of each indicator with HIV screening was estimated by its aOR and 95% CI, obtained from a multiple multinomial logit model applied to seven imputed datasets. Each dataset contained 13,210 pregnant women.

The odds of an unscreened outcome was significantly higher in women in a legally registered partnership (OR: 1.3; 95% CI: 1.1–1.6), 1–2 years of post-secondary schooling (OR: 1.6; 95% CI: 1.2–2.1) and part-time employment (OR: 1.4; 95% CI: 1.1–1.8). The odds of a missing information outcome was higher among multiparous women (OR: 1.4; 95% CI: 1.2–1.5).

Women with inadequate antenatal care were more likely to be unscreened for HIV (OR: 1.9; 95% CI: 1.5–2.4) than those whose antenatal care was adequate.

The odds of an unscreened outcome was significantly lower for women cared for by an obstetrician/gynaecologist in private practice (OR: 0.7; 95% CI: 0.5–0.9) but higher for women receiving care from more than one healthcare provider (OR: 1.8; 95% CI: 1.1–2.8) than any of the others. Information about HIV screening was missing more often in women cared for by an obstetrician/gynaecologist in private practice (OR: 1.3; 95% CI: 1.1–1.5) or a GP (OR: 1.4; 95% CI: 1.1–1.9) than those cared for by the other healthcare providers.

The odds of an unscreened outcome was significantly higher for women in the Grand-Est, Bretagne, Centre, and Pays de la Loire regions, and lower for those in the Normandie and Overseas regions. Similarly, the odds of a missing information outcome was higher for women in the Grand-Est and Pays de la Loire regions and lower for those in the Overseas region. It was also higher in the Nouvelle-Aquitaine, Bourgogne Franche-Comté and Occitanie regions.

Sensitivity analyses for multivariate regression performed on the complete pre-imputation database produced similar results (Supplementary Table S1).

## Discussion

This study shows a high rate of antenatal HIV screening among pregnant women living in France (96.8%). Screening resulted in the discovery of 19 new cases of HIV infection among women tested during pregnancy (0.15%), even though some pregnant women were not offered or refused the opportunity for antenatal HIV testing (1.0%) or their medical records had no information on the performance or the result of a HIV test (2.2%).

Among high-income countries, only the Netherlands [7] has previously evaluated its universal antenatal HIV screening programme. The proportion of HIV screening among women who gave birth in the Netherlands in 2006 to 2008 was higher than in France in 2016 (99.8% vs 96.8%). This difference is likely because of the use of different data sources; the Dutch study collected information about antenatal HIV testing from a national electronic database while ours was based on the use of medical records with a relatively high rate of missing information about HIV testing (2.2%).

The practical effectiveness of the French antenatal HIV testing programme is demonstrated by the absence of significant associations between HIV screening and maternal country of birth, single motherhood, household income or health insurance plan. Nonetheless, some women still receive inadequate antenatal care, which was associated with higher odds of not being screened. This finding is similar to that of Breese et al. who concluded that a lack of HIV screening was associated with a lack of antenatal care in women who gave birth between 1998 and 2001 in a Colorado, US hospital [15]. This association was also found in the Lazio region, Italy by Valle et al. [11]. In France, women with inadequate antenatal care were found to be principally women who made their first antenatal visit too late, after the first trimester of pregnancy [19]. Inadequate care is more frequent in women who are younger, multipara, migrant, single, living in deprived neighbourhoods, do not have health insurance or have a low educational level [19,20].

Our results also showed that women in a legally registered partnership or with 1–2 years of post-secondary education or with part-time employment had significantly higher odds of not being screened. Several hypotheses might explain these results.

Women in legally registered partnerships might be considered at low risk of HIV infection on the assumption that they only have one sexual partner. Healthcare providers might therefore not routinely offer them HIV screening.

The higher odds observed for no testing in women with an intermediate education level could be explained by differences in health literacy and inequities in care. Healthcare providers may think, for example, that the risk of HIV exposure for women decreases with increasing education level and thus women with a lower educational may be prescribed HIV testing more often as a result; these women may also be more closely followed up more to ensure that they are tested. Indeed, educational disparities can impair low-educated women’s knowledge or ability to engage in preventive health behaviours leaving them vulnerable to HIV and other diseases [21]. Conversely, highly educated women, i.e. those with 3 or more years post-secondary schooling, may have a greater awareness and understanding of the testing recommendations and therefore be more likely to adhere to them. In comparison with low and high-educated women, women with an intermediate level of education level may have less attention from health professionals about HIV testing and less ability to understand the importance of it.

In terms of an explanation for a significantly higher odds of unscreened outcomes for women working part-time, we suggest that socioeconomic factors might have an indirect influence even though being unscreened was not significantly associated with the mother’s household income in our multivariate analyses. In a report about part-time employed women, the French Economic, Social and Environmental Council [22] revealed that part-time employment was more common in women with a low education level, a low-skill job or those living in rural areas. Women with part-time employment may thus have more restrictions on their outcome and the organisation of their pregnancy follow-up, which could reduce their opportunities to access HIV testing. Furthermore, part-time employment is a factor that increased socioeconomic inequalities as it is not a personal choice for one-third of women [22].

The higher odds of missing information about HIV screening in the medical records of multiparous women might be because of providers assuming that a test was performed in a previous pregnancy and that nothing has changed.

The odds of not being screened for-HIV was notably higher for women with more than one care provider in the first 6 months of pregnancy. This may not only suggest a lack of HIV screening but also of follow-up antenatal care among these women. A significantly higher odds of missing information about HIV testing was also found among women receiving antenatal care from GPs or obstetricians/gynaecologists in private practice during the first 6 months of pregnancy. These women only booked appointments in maternity units for delivery after the sixth month of pregnancy and the results of their earlier antenatal examinations should have been transferred. However, HIV test information might have been lost because of the transfer and not checked for at the maternity unit. Our findings should alert obstetrics professionals to think about HIV testing at each antenatal visit, especially for women with late booking in maternity units for delivery. Moreover, all maternity units should make sure they collect and record the result of any HIV tests in the medical record of all women.

HIV infection rates differ between areas. In France, the rate of people newly diagnosed with HIV in 2015 was substantially higher in the Paris region (208 per million inhabitants) and the Overseas region (214 per million) than in the other regions (between 28 and 85 per million) [23]. In this epidemiological context and consistent with French HIV screening recommendations, the HIV testing rates are higher among the general population of people from high-prevalence regions [16]. Thus, the highest proportion of screened women was in those who gave birth in the Overseas and Paris regions or those born in sub-Saharan Africa (98.4%).

Our results appear robust after sensitivity analyses. Additionally, antenatal HIV screening information was collected from medical records in 2016 and is thus more reliable than that collected from interviews in previous years [13]. This study’s use of data from the nationally representative sample of pregnant women in France in 2016 is its principal strength.

Nonetheless, our study still presents several limitations. First, we did not know the exact proportion of HIV test refusals because the reasons for being unscreened were not documented. Moreover, for unscreened women with a recent negative HIV test result before pregnancy, we neither know when this test was performed nor if they refused a new one or if a new one was not offered. Furthermore, as the date of HIV screening during pregnancy was not available in 2016 NPS, we did not know if HIV testing was performed during the first trimester or later or repeated.

In conclusion, our study shows inadequate antenatal care as an important indicator for HIV screening and multipara as a risk factor for missing information on HIV testing. Our results also suggest that women perceived as low risk by providers, i.e. legally registered partnerships, were less likely to be screened for HIV while screening is well performed among women who were born outside France, who were unemployed or who had a low income. Although some women do not receive or take advantage of this opportunity, our findings demonstrate that the antenatal HIV screening programme is effective in detecting HIV among pregnant women in France, i.e. has high coverage and yield, and that antenatal HIV screening should continue to be carried out for all pregnant women: healthcare providers should systematically propose HIV testing for all women during the first trimester of pregnancy, and at least propose a repeat test at the beginning of the third trimester for women at high risk. Finally, to improve our understanding of why sometimes HIV screening is not performed and why there is no information on the performance or result of a HIV test in women’s medical records, further studies of the attitudes and practices of healthcare providers about antenatal HIV screening in relation to maternal characteristics would be beneficial.

## Supplementary Data

241000 Supplementary Table S1
