# Supplementary material for: Antenatal HIV screening: results from the National Perinatal Survey, France, 2016
Source: Euro Surveill. 2019 Oct 3;24(40):1800573. doi: 10.2807/1560-7917.ES.2019.24.40.1800573 (PMC6784449; doi:10.2807/1560-7917.ES.2019.24.40.1800573)
Supplement: Supplementary Table S1 [file 1800573_LOT_SupplementaryTableS1.pdf]

Supplementary Table S1

Disclaimer: This supplementary material is hosted by Eurosurveillance as supporting information alongside the article “Antenatal HIV screening: results from the National Perinatal Survey, France, 2016” on behalf of the authors who remain responsible for the accuracy and appropriateness of the content. The same standards for ethics, copyright, attributions and permissions as for the article apply. Supplements are not edited by Eurosurveillance and the journal is not responsible for the maintenance of any links or email addresses provided therein.

Table S1. Adjusted odds ratios of being unscreened for HIV and missing information about an HIV test, results obtained from sensitivity analyses performed in the complete pre-imputation database, National Perinatal Survey, France, 2016 (n = 11,751)

		HIV screening outcome[a]	
**Variable**		**Unscreened vs screened**	**Missing information vs screened**
		aOR (95% CI)	aOR (95% CI)
**Legally registered partnership**	**No**	Reference	Reference
	**Yes**	**1.40 (1.16-1.69)**	0.90 (0.80-1.02)
**Education level**	**Did not complete high school**	1.01 (0.72-1.42)	0.74 (0.58-0.94)
	**Completed high school**	0.91 (0.65-1.28)	1.00 (0.81-1.24)
	**1-2-years post-secondary school**	**1.64 (1.22-2.19)**	1.04 (0.84-1.29)
	**3-4-years post-secondary school**	0.76 (0.52-1.12)	1.02 (0.81-1.28)
	**≥ 5-years post-secondary school**	Reference	Reference
**Maternal occupational status**	**Unemployed**	Reference	Reference
	**Employed part-time**	**1.59 (1.24-2.04)**	1.07 (0.88-1.29)
	**Employed full-time**	0.79 (0.63-1.00)	0.97 (0.82-1.13)
**Parity**	**Primiparous**	Reference	Reference
	**Multiparous**	1.12 (0.94-1.34)	1.43 (1.26-1.61)
**Main healthcare provider**	**Obstetrician/gynaecologist (private)**	**0.62 (0.46-0.84)**	**1.36 (1.07-1.73)**
	**Obstetrician/gynaecologist (public)**	1.11 (0.78-1.58)	0.96 (0.70-1.32)
	**General practitioner**	1.11 (0.71-1.73)	**1.62 (1.16-2.25)**
	**Midwife (public)**	Reference	Reference
	**Midwife (private)**	0.91 (0.58-1.44)	1.17 (0.82-1.66)
	**More than one healthcare provider**	**2.54 (1.46-4.45)**	0.65 (0.28-1.50)
**Inadequate antenatal care**	**No**	Reference	Reference
	**Yes**	**1.78 (1.31-2.41)**	1.29 (0.98-1.70)
**Location of maternity units**	**Paris region[b]**	Reference	Reference
	**Grand-Est**	**2.31 (1.43-3.71)**	**1.86 (1.30-2.65)**
	**Nouvelle-Aquitaine**	1.10 (0.58-2.09)	**2.01 (1.42-2.86)**
	**Bourgogne-Franche-Comté**	0.57 (0.18-1.76)	**2.07 (1.30-3.28)**
	**Bretagne**	**3.14 (1.90-5.18)**	1.12 (0.67-1.86)
	**Centre-Val-de-Loire**	**3.42 (2.01-5.84)**	1.09 (0.61-1.96)
	**Occitanie**	0.97 (0.49-1.92)	**2.01 (1.42-2.84)**
	**Hauts-de-France**	0.78 (0.42-1.48)	0.98 (0.65-1.48)
	**Normandie**	**0.19 (0.04-0.93)**	0.79 (0.44-1.42)
	**Provence-Alpes-Côte d’Azur[c]**	1.47 (0.82-2.61)	0.83 (0.50-1.38)
	**Pays de la Loire**	**3.91 (2.54-6.03)**	**5.23 (3.93-6.95)**
	**Auvergne-Rhône-Alpes**	1.04 (0.63-1.74)	1.16 (0.83-1.64)

	**Overseas[d]**	**0.32 (0.10-1.02)**	**0.09 (0.02-0.43)**

CI: confidence interval; aOR: adjusted odds ratio.

[a] Screened (n = 11,384); unscreened (n = 112); missing information (n = 255).

[b] The Île-de-France region is known as the Paris region.
[c] The region of Corsica is included in the Provence-Alpes-Côte d'Azur region.
[d] Guadeloupe, Guyana, Martinique, Mayotte and La Réunion are regrouped in the Overseas region.
The association of each indicator with HIV screening was estimated by its aOR and 95%CI.
